# Distributed parameter model of dynamic contrast-enhanced MRI in the identification of IDH mutation, 1p19q codeletion, and tumor cell proliferation in glioma patients

**DOI:** 10.3389/fonc.2024.1333798

**Published:** 2024-10-25

**Authors:** Kai Zhao, Huiyu Huang, Eryuan Gao, Jinbo Qi, Ting Chen, Gaoyang Zhao, Guohua Zhao, Yu Zhang, Peipei Wang, Jie Bai, Yong Zhang, Zujun Hou, Jingliang Cheng, Xiaoyue Ma

**Affiliations:** ^1^ Department of Magnetic Resonance Imaging, the First Affiliated Hospital of Zhengzhou University, Zhengzhou, China; ^2^ Jiangsu Key Laboratory of Medical Optics, Suzhou Institute of Biomedical Engineering and Technology, Chinese Academy of Sciences, Suzhou, China

**Keywords:** glioma, dynamic contrast-enhanced MRI, distributed parameter model, IDH mutation, *1p/19q* codeletion, Ki-67

## Abstract

**Objectives:**

To investigate the clinical value of hemodynamic parameters derived from dynamic contrast-enhanced MRI (DCE-MRI) in predicting glioma genotypes including isocitrate dehydrogenase (*IDH*) mutation, *1p/19q* codeletion status and the tumor proliferation index (*Ki-67*) noninvasively. And to compare the diagnostic performance of parameters of distributed parameter (DP)model and extended Tofts (Ex-Tofts) model.

**Materials and methods:**

Dynamic contrast-enhanced MRI (DCE-MRI) data of patients with glioma were prospectively enrolled from April 2021 to May 2023. The imaging data were analyzed using DP and Ex-Tofts model for evaluating the perfusion and permeability characteristics of glioma. Comparisons were performed according to *IDH* genotype in all glioma patients and *1p/19q* codeletion in *IDH* mutation glioma patients. Receiver operating characteristic (ROC) curves were generated for DCE-MRI parameters. The Spearman rank correlation coefficients were calculated between DCE MRI parameters and *Ki-67* index.

**Results:**

In *IDH*-mutation gliomas, a higher blood flow (F) was found in *1p/19q* codeletion gliomas than in *1p/19q* intact gliomas. No parameter derived from Ex-Tofts model showed significant differences in predicting *1p/19q* status. Fractional volume of interstitial space (*V*_e_) derived from both the DP and Ex-Tofts models exhibited optimal performance in predicting IDH genotype (AUC = 0.818, 0.828, respectively). *V*_e_ also showed the highest correlations with *Ki-67* LI within their respective models in all gliomas (*ρ* = 0.62, 0.61), indicating comparable moderate positive associations. *Ki-67*

**Conclusion:**

DP model showed a clear advantage in predicting *1p/19q* status compared to Ex-Tofts model. The DP and Ex-Tofts models performed similarly in predicting *IDH* mutation and *Ki-67* index.

## 1 Introduction

Gliomas, being the most commonly occurring primary malignant brain tumors in adults (1), are classified by the 2021 version of the World Health Organization (WHO) into three groups based on two critical molecular markers: the isocitrate dehydrogenase (*IDH*) genotype and *1p/19q* codeletion status. The groups include *IDH* wild-type, *IDH* mutation with *1p/19q* intact, and *IDH* mutation with *1p/19q* codeletion (2). This new classification system applies to the glioma subtype, thus establishing a link between the grade of glioma and not just its natural disease progression but also the impact of clinical treatment on the course and prognosis of the disease. *Ki-67*, a nuclear antigen involved in cellular proliferation, represents a valuable biomarker for the evaluation of cell proliferation. An elevation in *Ki-67* labeling index (LI) indicates augmented tumor proliferation, which in turn correlates with inferior prognosis among glioma patients (3). Studies have demonstrated that certain genetic factors, including *IDH* mutation, *1p/19q* codeletion, and o6-methylguanine-DNA-methyltransferase (*MGMT*) promoter methylation, can predict treatment response, particularly in the context of chemotherapy (4, 5). Moreover, in recent years, additional treatment modalities, such as targeted therapy and radioimmunotherapy, have emerged and are currently under investigation in clinical trials (6, 7). These innovative approaches rely on the identification of specific molecular targets within glioma cells, highlighting the significance of genetic molecular diagnosis in guiding treatment decisions and identifying suitable targets for these therapies.

Therefore, the histological diagnosis and gene molecular diagnosis of glioma play a pivotal role in developing personalized preoperative treatment strategies, and have substantial implications in improving patients’ quality of life and prognosis. Currently, histopathological analysis based on resection or biopsy is considered the most reliable means for molecular diagnosis of glioma genes (8). However, it is characterized by its high cost, demanding expertise, and the risk of sampling errors (9). Particularly in patients unsuitable for surgery, obtaining necessary pathological information without increasing patient burden and risk can maximize their benefits. Against this backdrop, many radiologists are actively exploring the relationship between imaging techniques and molecular biomarkers, aiming to predict molecular information non-invasively (10).

Dynamic contrast-enhanced magnetic resonance imaging (DCE-MRI) is a technique employed to assess blood-brain barrier (BBB) disruption and neovascularization in gliomas. These characteristics offer essential insights into the tumor microenvironment and metabolic properties of various glioma subtypes (11). Several recent reviews (12–14) have collectively concluded that while DCE imaging exhibits promising clinical application prospects in predicting *IDH* status, it lacks satisfactory performance in identifying *1p/19q* codeletion, and further research is still needed to investigate the use of DCE imaging in predicting *1p/19q* status. In DCE-MRI, mathematical models are employed to estimate pharmacokinetic parameters that provide insights into the perfusion and permeability of lesions. The accurate characterization of these parameters relies on an appropriate mathematical model. Presently, the extended Tofts (Ex-Tofts) model is widely used in DCE-MRI due to its relatively relaxed requirements for equipment and scan duration (15). However, the main parameter, transfer constant (*K* ^trans^), in Ex-Tofts model does not accurately reflect vascular permeability since it does not differentiate between the intravascular transport of tracer molecules and the exchange process of tracer molecules between the intravascular and interstitial spaces (16). As technology and equipment continue to advance, the distributed parameter (DP) model was proposed to addresses such limitation by separately considering the intravascular transport and the exchange between the intravascular and interstitial compartments (17). DP model incorporates two key parameters: blood flow (F), which characterizes intravascular transport, and the permeability-surface area product (PS), which describes the exchange process.

In this study, our objective was to evaluate the potential of DCE-MRI using the DP model in predicting the *IDH* genotype, chromosome *1p/19q* codeletion status, and *Ki-67* LI in adult diffuse gliomas, and to assess whether the DP model offers advantages in the molecular diagnosis of glioma, which may enhance their clinical management.

## 2 Materials and methods

This retrospective study was approved by our hospital’s institutional review board, and informed consent was waived.

### 2.1 Study participants

Patients with glioma who underwent DCE examination between April 2021 and May 2023 were retrospectively collected. The inclusion criteria were as follows: DCE-MRI performed within two weeks prior to surgery and before the initiation of antitumor therapy, and a diagnosis of gliomas of grade 2-4 based on the 2021 WHO guideline on brain tumor classification following tumor resection and pathology examination. The exclusion criteria were: a diagnosis of WHO grade 1 glioma; inadequate MRI quality. The *IDH*1/2 mutations in the hotspot codons R132 and R172 on the excised surgical specimens were determined by Sanger sequencing or immunohistochemical staining. A mutation in any one of them was diagnosed as an *IDH* mutation. The *1p/19q* deletions were detected through fluorescence *in situ* hybridization analysis. The *Ki-67* labeling index was determined by using immunohistochemistry.

### 2.2 MR imaging acquisition

All scans were conducted using a 3.0 T MRI scanner from Siemens Healthcare (Magnetom Prisma). The DCE scan employed an axial fast-spoiled gradient (SPGR) echo sequence. This sequence included a pre-contrast and a post-contrast phase with the following parameters: TR/TE (3.03 ms/1.06 ms), FOV (230 × 230mm^2^), matrix (192×134.4), slice thickness (5 mm), flip angles for the pre-contrast scan (3°, 6°, and 9°), and for the post-contrast scan (9°). For each flip angle, ten dynamic pre-contrast scans were acquired, while the post-contrast sequence consisted of 180 dynamic scans, with a temporal resolution of 2 seconds. The contrast agent used was Gadovist (Magnevist; Bayer Schering Pharma AG), administered at an injection rate of 3.5 mL/sec (followed by a 20 mL normal saline flush), with a dose of 0.1 mmol/kg body weight.

### 2.3 Image processing

DCE images were processed using a commercially software (MItalytics, FITPU Healthcare, Singapore). Two experienced neuroradiologists (K.Z. and X.M., with 3 and 11 years of experience, respectively) manually delineated the tumor region of interest (ROI) in reference to the late-phase dynamic T1-enhanced image (with obvious enhanced lesions) or the T2-FLAIR sequence images (without obvious enhanced lesions). The delineation includes the solid components of the tumor and avoids areas of necrosis, hemorrhage, calcification, large vessels, and cystic regions. Voxels in ROI were aggregated, and the median values of following kinetic parameters were calculated for each patient: Ex-Tofts model derived transfer constant *K* ^trans^ (min^−1^), fractional volume of extravascular extracellular space *V*_e_ (mL/100 mL), plasma fractional volume *V*_p_ (mL/100 mL), efflux rate constant *K*_ep_ (min^−1^). DP model derived blood flow F (mL/min/100 mL), permeability-surface area product PS (mL/min/100 mL), extraction ratio of first pass E (%), *V*_e_ and *V*_p_ (same as in the Ex-Tofts model). To ensure completeness, the operational equations of these models, which specify the relationship between tissue tracer concentration C_tiss_(t) (as a function of time t) and AIF as well as relevant physiological parameters, are presented below:

Ex-Tofts model:


(1) $${\text{C}}_{\text{tiss}}\left(t\right)={\text{AIF }}_{\text{vp}}+\text{AIF}⊗{\text{ K}}^{\text{trans}}\exp\left(-\frac{{\text{K}}^{\text{trans}}}{V_{\text{e}}}t\right)$$

DP model:


(2) $$\begin{array}{c} {\text{C}}_{\text{tiss}}\left(t\right)=AIF⊗\\ {\text{F}}_{p}\left\{\begin{array}{c} u\left(t\right)-u\left(t-\frac{v_{p}}{F_{p}}\right)+\\ u\left(t-\frac{v_{p}}{F_{p}}\right)\left\{1-\exp\left(-\frac{PS}{F_{p}}\right)\left[1+\overset{\text{t-}\frac{{\text{v}}_{p}}{{\text{F}}_{p}}}{\underset{0}{\int }}\;\exp\left(-\frac{\text{PS}}{{\text{v}}_{e}}\tau \right)\sqrt{\frac{\text{PS}}{{\text{v}}_{e}}\frac{\text{PS}}{F_{p}}\frac{1}{\tau }}{\text{I}}_{1}\left(2\sqrt{\frac{\text{PS}}{{\text{v}}_{e}}\frac{\text{PS}}{F_{p}}\tau }\right)\text{d}\tau \right]\right\} \end{array}\right\} \end{array}$$

### 2.4 Statistical analysis

Statistical analysis was performed using R software (version 4.3.1; https://www.R-project.org/). Normality of data and homogeneity of variance were assessed using Shapiro-Wilk and Levene’s tests, respectively. Differences in parameters and mean age were evaluated between *IDH*-mutation and *IDH*-wild-type gliomas, as well as *IDH* mutation&*1p/19q* intact and *IDH* mutation&*1p/19q* codeletion gliomas using independent t-test or Mann–Whitney U test according to the results of test for normality and homoscedasticity. Benjamini-Hochberg correction was applied to adjust the *P* values of DCE parameters for multiple comparisons. The receiver operating characteristic (ROC) curves were utilized for assessing the performance of kinetic parameters in predicting *IDH* mutation and *1p/19q* status. The diagnostic performance was quantified using the area under the ROC curve (AUC). The DeLong test was conducted to compare the diagnostic performance of the Ex-Tofts model and the DP model by comparing their respective parameters with the largest AUC values in each model. The method of Youden index was utilized to determine the optimal threshold for classification and compute the corresponding sensitivity, specificity, and accuracy. Relationship between Ex-Tofts parameters, DP parameters and *Ki-67* LI was assessed using the Spearman correlation test. Statistical significance was set at *P*< 0.05.

## 3 Results

### 3.1 Patient characteristics

48 glioma patients were finally included in the study. **Table 1** summarizes the clinical, demographic, and pathological characteristics of the patients. Based on the 2021 WHO classification of CNS tumors, the tumors were classified into *IDH*-mutation and *1p/19q* intact glioma (WHO grade 2 astrocytoma, n=3; WHO grade 3 astrocytoma, n=3; WHO grade 4 astrocytoma, n=3), *IDH*-mutation and *1p/19q* codeletion glioma (WHO grade 2 oligodendroglioma, n=7; WHO grade 3 oligodendroglioma, n=8), and *IDH*-wild-type glioma (WHO grade 4 glioblastoma, n=24). Patients with *IDH* wild-type glioma were found to be older than those with *IDH*-mutation glioma. There was no significant difference between glioma subtypes in terms of sex distribution.

**Table 1:** Clinical and demographic data of the study cohort.

	Male	Female	Age (years)	P Value of Sex	P Value of Age
*IDH* mutation	17	7	44 ± 9	0.079	0.004
*IDH* wild-type	11	13	53 ± 12		
*IDH* mutation&*1p/19q* intact	7	2	42 ± 10	0.144	0.346
*IDH* mutation&*1p/19q* codeleted	10	5	46 ± 9		

### 3.2 Kinetic parameters in identification of molecular subtypes

**Figure 1:**
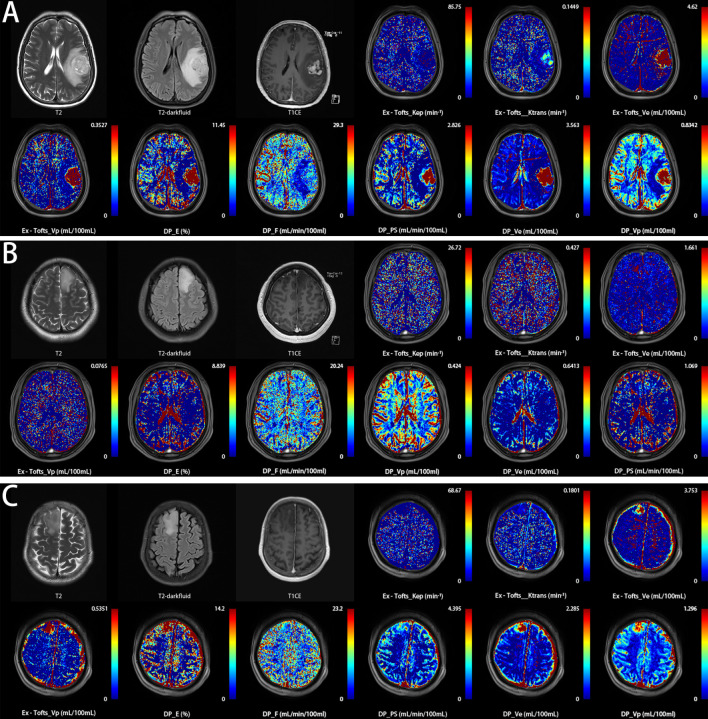
Three representative patients with glioma were correctly classified into their respective subtypes based on the threshold values of DCE parameters in this study, using pathological examination results as the gold standard. **(A)** a 59-year-old female with histologically proven glioblastoma IDH wild-type (Ex-Tofts_*V*_e_ = 16.08; DP_F = 9.21). **(B)** a 46-year-old male with histologically proven astrocytoma IDH mutation&*1p/19q* intact (Ex-Tofts_*V*_e_ = 0.08; DP_F = 7.00). **(C)** a 47-year-old female with histologically proven oligodendroglioma IDH mutation&*1p/19q* codeleted (Ex-Tofts_*V*_e_ = 1.34; DP_F = 8.82).

**Figure 2:**
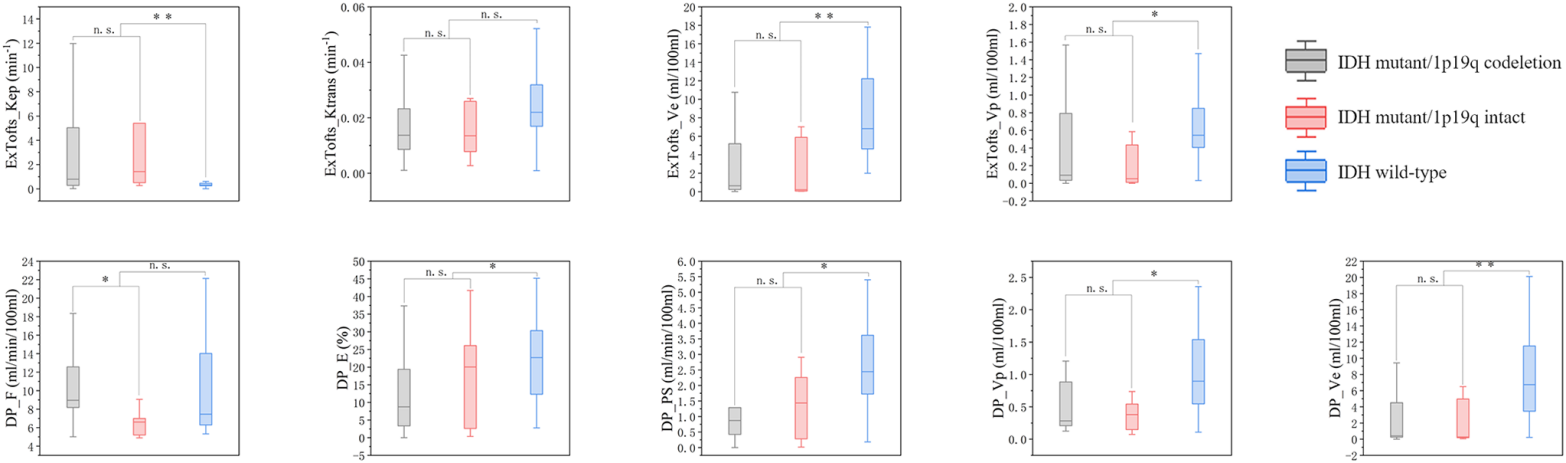
Boxplots of kinetic parameters in differentiating three types of gliomas, n.s. stands for not significant, *P< 0.05, **P< 0.01, ***P< 0.001.

As the distribution of all data did not meet the criteria for normality according to the Shapiro-Wilk test at a significance level of 5%, the Mann-Whitney U test was used to assess the differences between parameters. *K*_ep_ derived from Ex-Tofts model was found significantly higher in *IDH* mutation gliomas than in *IDH* wild-type gliomas. *V*_e_, *V*_p_ derived from Ex-Tofts model and *V*_e_, *V*_p_, PS, E derived from DP model were found significantly lower in *IDH* mutation gliomas compared to *IDH* wild-type gliomas (**Table 2**). Only the F derived from DP model exhibited a significant difference between *1p/19q* codeleted glioma and *1p/19q* intact glioma, and the *1p/19q* codeleted glioma had a higher F value compared to the *1p/19q* intact glioma. No parameters in Ex-Tofts showed significant differences in predicting *1p/19q* status (**Table 3**). Representative cases of three different subtypes glioma are shown in **Figure 1**. **Figure 2** shows the boxplots of Ex-Tofts and DP parameters, illustrating the intergroup differences in the distribution of kinetic parameters.

**Table 2:** Results of kinetic parameters in predicting *IDH* genotype.

	*IDH* Mutation	*IDH* Wild-type	U	P
Ex-Tofts_ *K* ^trans^	0.014 (0.008,0.024)	0.022 (0.017,0.032)	201	0.149
Ex-Tofts_ *V*_e_	0.633 (0.214,5.370)	6.825 (4.712,12.221)	99	< 0.001^*^
Ex-Tofts_ *V*_p_	0.078 (0.026,0.473)	0.544 (0.444,0.831)	149	0.011^*^
Ex-Tofts_ *K*_ep_	0.926 (0.466,5.069)	0.31 (0.254,0.446)	452	0.003^*^
DP_F	8.532 (6.569,10.002)	7.454 (6.308,13.777)	272	0.866
DP_ *V*_p_	0.345 (0.206,0.590)	0.897 (0.600,1.508)	158	0.017^*^
DP_ *V*_e_	0.415 (0.235,4.625)	6.739 (3.558,11.505)	105	< 0.001^*^
DP_PS	0.896 (0.356,2.241)	2.445 (1.769,3.527)	143	0.009^*^
DP_E	9.400 (3.092,20.535)	22.696 (12.670,30.283)	144	0.009^*^

^*^P< 0.05.

**Table 3:** Results of kinetic parameters in predicting *1p/19q* status.

	*1p/19q* intact	*1p/19q* codeleted	U	P
Ex-Tofts_ *K* ^trans^	0.014 (0.008,0.026)	0.014 (0.010,0.021)	68	> 0.99
Ex-Tofts_ *V*_e_	0.217 (0.076,5.887)	0.643 (0.249,4.365)	83	0.669
Ex-Tofts_ *V*_p_	0.053 (0.012,0.435)	0.093 (0.036,0.600)	81	0.669
Ex-Tofts_ *K*_ep_	1.427 (0.503,5.400)	0.798 (0.397,4.298)	54	0.669
DP_F	6.607 (5.196,6.997)	8.963 (8.32,12.418)	107	0.040^*^
DP_ *V*_p_	0.380 (0.149,0.542)	0.283 (0.215,0.679)	73	0.866
DP_ *V*_e_	0.276 (0.182,4.969)	0.415 (0.247,3.201)	76	0.823
DP_PS	1.437 (0.281,2.257)	0.872 (0.457,1.245)	67	> 0.99
DP_E	20.057 (2.636,26.107)	8.728 (3.394,15.575)	59	0.823

^*^P< 0.05.

### 3.3 ROC curve analysis

**Figure 3:**
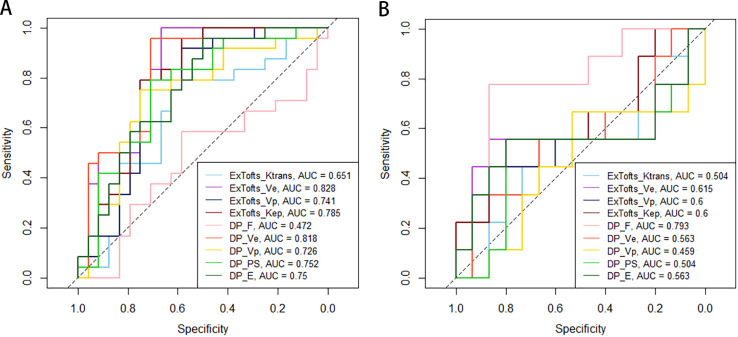
Receiver operating characteristic (ROC) plots and areas under ROC curve (AUCs) of Ex-Tofts and DP model parameters in differentiating of *IDH* mutation status **(A)** and *1p/19q* codeletion status **(B)**.

**Tables 4** and **5** respectively summarizes the results of ROC curve analysis in differentiating *IDH* mutation (mutation vs. wild-type) and *1p/19q* codeletion status in *IDH* mutation glioma (intact vs. codeleted). *V*_e_ attained the best performance in discriminating *IDH*-mutation from *IDH*-wild-type gliomas in both Ex-Tofts and DP model (AUC = 0.828 and 0.818, respectively). Delong test showed no significant difference between the AUCs of above two parameters (*z* = 0.509, *P* = 0.611). Among DP-derived parameters, F showed a good performance in predicting *1p/19q* status with AUC = 0.793. The plots of ROC curves are shown in **Figure 3**.

**Table 4:** ROC Analysis of kinetic parameters with significant difference in predicting *IDH* genotype.

	AUC (95%CI)	P	SEN	SPC	ACC	Cut-off
Ex-Tofts_ *K* ^trans^	0.651 (0.488, 0.814)	0.035	0.583	0.792	0.688	0.016
Ex-Tofts_ *V*_e_	0.828 (0.706, 0.950)	< 0.001	0.667	1	0.833	1.670
Ex-Tofts_ *V*_p_	0.741 (0.591, 0.891)	< 0.001	0.583	0.917	0.750	0.200
Ex-Tofts_ *K*_ep_	0.785 (0.646, 0.923)	< 0.001	0.583	0.958	0.771	0.640
DP_F	0.472(0.302,0.642)	0.626	0.417	0.417	0.417	7.863
DP_ *V*_p_	0.726 (0.574, 0.877)	0.002	0.750	0.750	0.750	0.600
DP_ *V*_e_	0.818 (0.691, 0.945)	< 0.001	0.708	0.958	0.833	1.925
DP_PS	0.752 (0.608, 0.895)	< 0.001	0.708	0.792	0.750	1.535
DP_E	0.750 (0.609, 0.891)	< 0.001	0.500	0.958	0.729	8.805

**Table 5:** ROC Analysis of kinetic parameters with significant difference in predicting *1p/19q* status.

	AUC (95%CI)	P	SEN	SPC	ACC	Cut-off
Ex-Tofts_ *K* ^trans^	0.504(0.245,0.762)	0.489	0.733	0.333	0.583	0.010
Ex-Tofts_ *V*_e_	0.615(0.344,0.885)	0.203	0.867	0.556	0.75	0.222
Ex-Tofts_ *V*_p_	0.600(0.342,0.858)	0.223	0.867	0.444	0.708	0.022
Ex-Tofts_ *K*_ep_	0.600(0.350,0.850)	0.216	0.667	0.556	0.625	1.342
DP_F	0.793 (0.595, 0.99)	0.002	0.867	0.778	0.833	7.154
DP_ *V*_p_	0.459(0.194,0.724)	0.618	0.467	0.333	0.417	0.296
DP_ *V*_e_	0.563(0.303,0.823)	0.318	0.933	0.333	0.708	0.197
DP_PS	0.504(0.226,0.782)	0.490	0.200	0.444	0.292	1.363
DP_E	0.563(0.274,0.851)	0.334	0.800	0.556	0.708	19.717

### 3.4 Correlation of kinetic parameters with the Ki−67 LI

**Figure 4:**
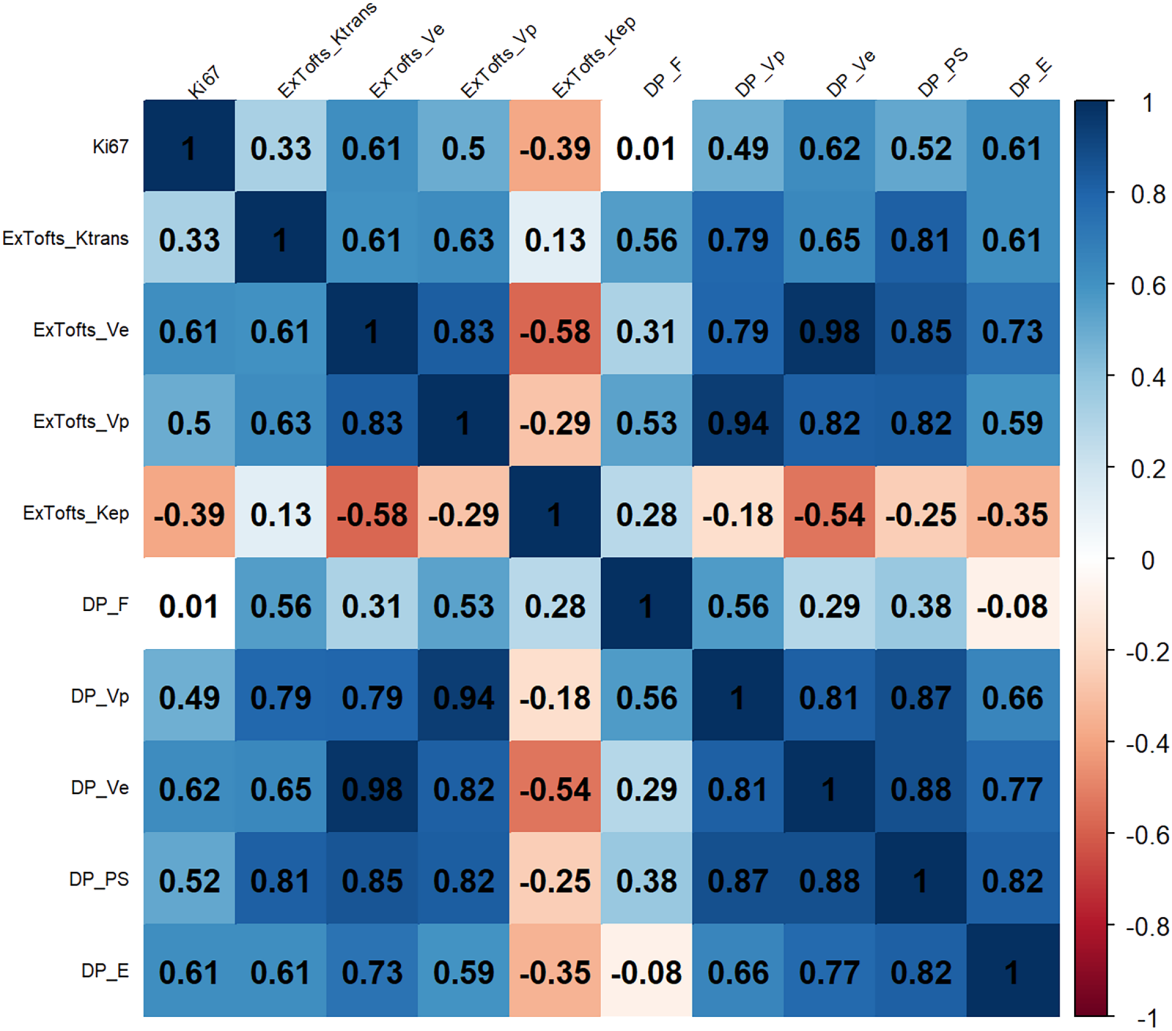
Heat map of correlations between the DCE parameters and *Ki-67* index.

The correlation results between the DCE parameters and *Ki-67* LI are shown in **Figure 4**. The corresponding *P* values are shown in the supplementary materials. *V*_e_ derived from DP model and the Ex-Tofts model was correlated best with *Ki-67* LI within their respective models in all gliomas with similar moderate positive correlations (*ρ* = 0.62, 0.61).

## 4 Discussion

This study aimed to investigate the potential of pharmacokinetic parameters derived from the Ex-Tofts model and the DP model as biomarkers for identifying *IDH* mutation, *1p/19q* codeletion status, and tumor cell proliferation (*Ki-67* LI) in gliomas. The results of this study revealed that there was no significant difference in the diagnostic efficacy between the two models for predicting *IDH* mutation status and *Ki-67* expression. In predicting the *1p/19q* status, the DP model demonstrated a substantial increase in the parameter F and exhibited favorable diagnostic performance (AUC = 0.793), while the Ex-Tofts model did not effectively predict the *1p/19q* status. This suggests that the DP model holds greater potential than the Ex-Tofts model in predicting the *1p/19q* status with the exclusive perfusion parameter F.

The measurement of F in predicting the *1p/19q* status was made possible by the DP model, which separately describe intravascular perfusion and exchange between the intravascular and extravascular spaces. These processes are characterized by two distinct parameters, namely F and PS. Conversely, the Ex-Tofts model combines these two processes into a single parameter, *K* ^trans^ (15). The use of appropriate pharmacokinetic models is crucial for the analysis of DCE-MRI data. Developing advanced pharmacokinetic models may be an important avenue to address the limitations of DCE in predicting *1p/19q* status. Higher F values observed in 2021 WHO oligodendrogliomas compared to astrocytomas may be related to their higher perfusion characteristics (18). An arterial spin labeling (ASL) study (19) has revealed that the cerebral blood flow (CBF) is significantly higher in oligodendrogliomas than astrocytomas, attributed to higher vascular density and gray matter involvement in oligodendrogliomas. Although CBF in ASL and F in DCE are not completely comparable, changes in this hemodynamic parameter indicate that the high perfusion characteristics of oligodendrogliomas can be used to predict the *1p/19q* status, which corroborates our results. Another study (20) as also highlighted the higher perfusion characteristics of oligodendrogliomas compared to astrocytomas, utilizing dynamic susceptibility contrast-enhanced (DSC) MRI. This study indicated that oligodendrogliomas revealed significantly higher cerebral blood volume (CBV) when compared to astrocytomas. In DCE, the parameter *V*_p_ exhibits physiological similarity to CBV. *V*_p_ is a perfusion parameter that measures the fractional volume of the intravascular space and may be correlated with tissue microvascular density. Correlation analysis demonstrated that there was a relatively weak positive correlation between *V*_p_ and F (*ρ* = 0.56). This indicates that while both parameters represent tissue perfusion, they also possess a certain degree of independence from each other, suggesting that they characterize different aspects of tumor perfusion. Our results failed to found any significant difference in *V*_p_ between astrocytomas and oligodendrogliomas, which is consistent with Gupta’s (21) conclusion. However, Lee et al. (22) have found a significant increase in *V*_p_ in oligodendrogliomas. Currently, there is limited literature on the use of perfusion imaging for identifying *1p/19q* codeletion status in gliomas, and most studies focus on DSC-MRI (12). The role of DCE in predicting *1p/19q* codeletion status remains controversial, and selecting appropriate pharmacokinetic models may be crucial for improving its clinical utility. Our study suggested one of the limitations of the Ex-Tofts model in characterizing perfusion is its inability to describe tissue blood flow velocity, thus necessitating the development of advanced pharmacokinetic models that factor in the transport of contrast agent molecules within the vasculature.

In predicting the *IDH* genotype, both Ex-Tofts and DP models have existing research (23, 24), and our findings regarding the comparison of parameter magnitudes align with previous studies. We identified *V*_e_ as the most distinguishing feature in discriminating between *IDH*-mutation and *IDH*-wild-type gliomas. *V*_e_ refers to the fractional volume of the extravascular extracellular space. As tumor cells proliferate excessively, the interstitial space decreases, resulting in a smaller *V*_e_. Compared to *IDH* wild-type, *IDH* mutation could inhibit proliferation in glioma (25). However, unlike other solid tumors (16), a decrease in *V*_e_ suggests elevated vessel permeability rather than higher cell proliferation. The blood-brain barrier restricts the leakage of contrast agent molecules from the vasculature, leading to smaller measured *V*_e_ values. In *IDH* wild-type gliomas, we observed a significant increase in *V*_e_, indicating a greater tendency for contrast agent molecules to leak out. This can be attributed to the presence of newly formed immature blood vessels in *IDH* wild-type gliomas, along with the irregular arrangement of endothelial cells and detachment of pericytes and astrocytes from microvascular walls (26), which increase the permeability of the blood-brain barrier and promote microvascular leakage. Conversely, *IDH*-mutation gliomas have been shown to exhibit decreased activation of hypoxia-inducible factor 1*α* (HIF-1*α*), leading to a reduction in hypoxia-induced angiogenesis (27). DCE-MRI can indirectly predict these genetic alterations by describing changes in tissue permeability.

*Ki-67* LI showed the highest correlation coefficient with *V*_e_ of DP model among the DCE parameters with a moderate positive correlation observed (*ρ* = 0.62). The positive correlation between *V*_e_ and *Ki-67* may be related to the compromised integrity of the blood-brain barrier. The elevated proliferative activity of tumor cells requires a substantial amount of energy to sustain their rapid growth and division. In response to this increased energy demand, tumors activate various adaptive mechanisms, including the upregulation of HIF-1*α*, leading to an increase in tumor angiogenesis and a more abundant tumor microcirculation (28). The presence of newly formed and immature blood vessels increases tumor vascular permeability, facilitating the extravasation of contrast agents and subsequently resulting in elevated *V*_e_ values. This finding is consistent with Jiang et al. (29). However, we were unable to confirm a significant correlation between *K* ^trans^ and *Ki-67*, as they did. This discrepancy may be due to the fact that Jiang et al. measured the maximum values of tumor hemodynamic parameters, while we focused on the median values within the ROI. In future studies, we may consider employing histogram analysis of DCE data to further explore this correlation.

Several limitations should be acknowledged in our study. Firstly, the sample size was relatively small, potentially introducing chance correlations when predicting *1p/19q* status, and the single-center design mean that the thresholds we identified may not be generalizable to other centers, limiting their applicability. Therefore, a prospective study with a larger sample size and multi-center is warranted to validate these findings. Secondly, the ROI delineation in our study was manually performed, and the adoption of machine learning algorithms for automated delineation holds promise in improving the objectivity of our research. Lastly, due to the update of the 2021 WHO CNS glioma classification, glioma grading is now categorized within pathological subtypes. The sample size in our study cohort was insufficient to conduct predictive research on glioma grading. We plan to further expand the sample size to explore the role of various DCE models in predicting glioma grading in future research.

## 5 Conclusion

DP model provided additional information on blood flow rate compared to the Ex-Tofts model, and it demonstrated a clear advantage in predicting *1p/19q* status. However, it did not show a significant difference in predicting *IDH* and *Ki-67* compared to the Ex-Tofts model.

## Data Availability

The original contributions presented in the study are included in the article/supplementary material. Further inquiries can be addressed to the corresponding author.
